# Supplementary figures and images for: A biophysical minimal model to investigate age-related changes in CA1 pyramidal cell electrical activity
Source: PLoS One. 2024 Sep 4;19(9):e0308809. doi: 10.1371/journal.pone.0308809 (PMC11373847; doi:10.1371/journal.pone.0308809)

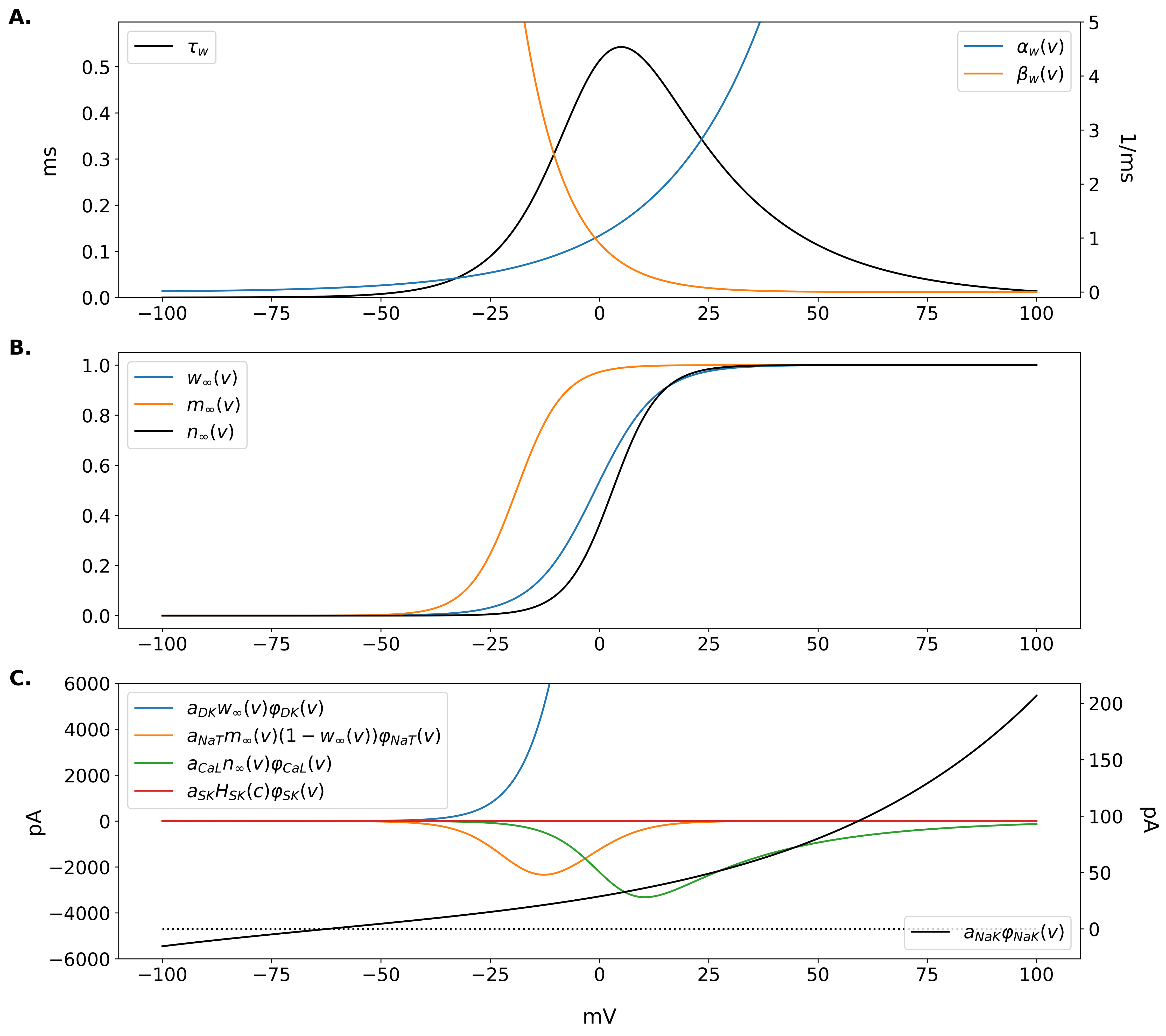

Supplement: S1 Fig — (A.) Forward (αw) and backward (βw) rate functions, and the time constant (τw), of activation of DK channels in the model. (B.) Steady-state activation curves for DK (w∞), Na+(m∞), and L-type Ca2+(n∞) channels. (C.) Full expressions and plots for each ion current in the model. (TIF) [file pone.0308809.s001.tif]

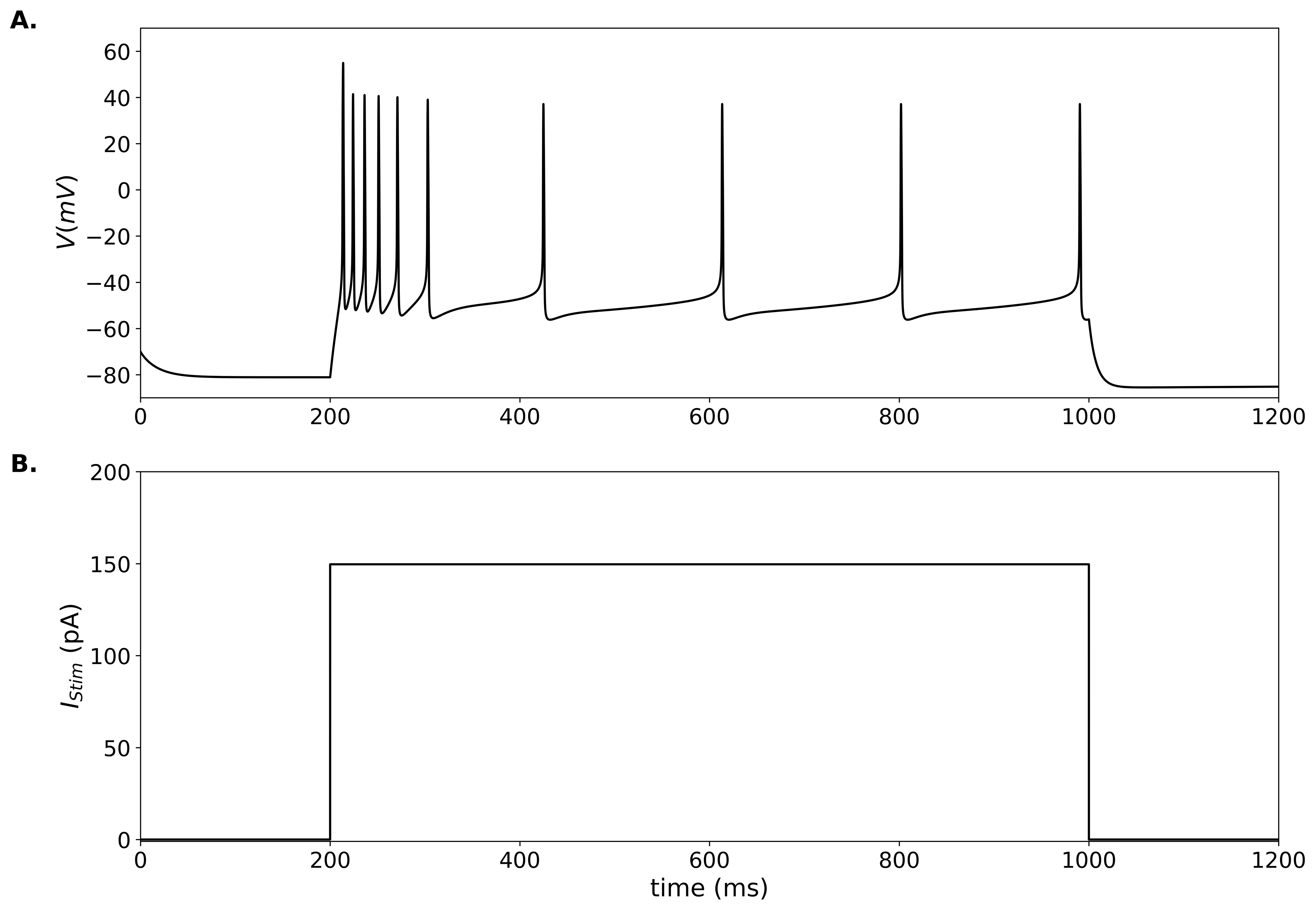

Supplement: S2 Fig — (A.) Voltage response of the young model PC (yPC) in response to a 800 ms 150 pA square-pulse stimulation seen in (B.). (TIF) [file pone.0308809.s002.tif]

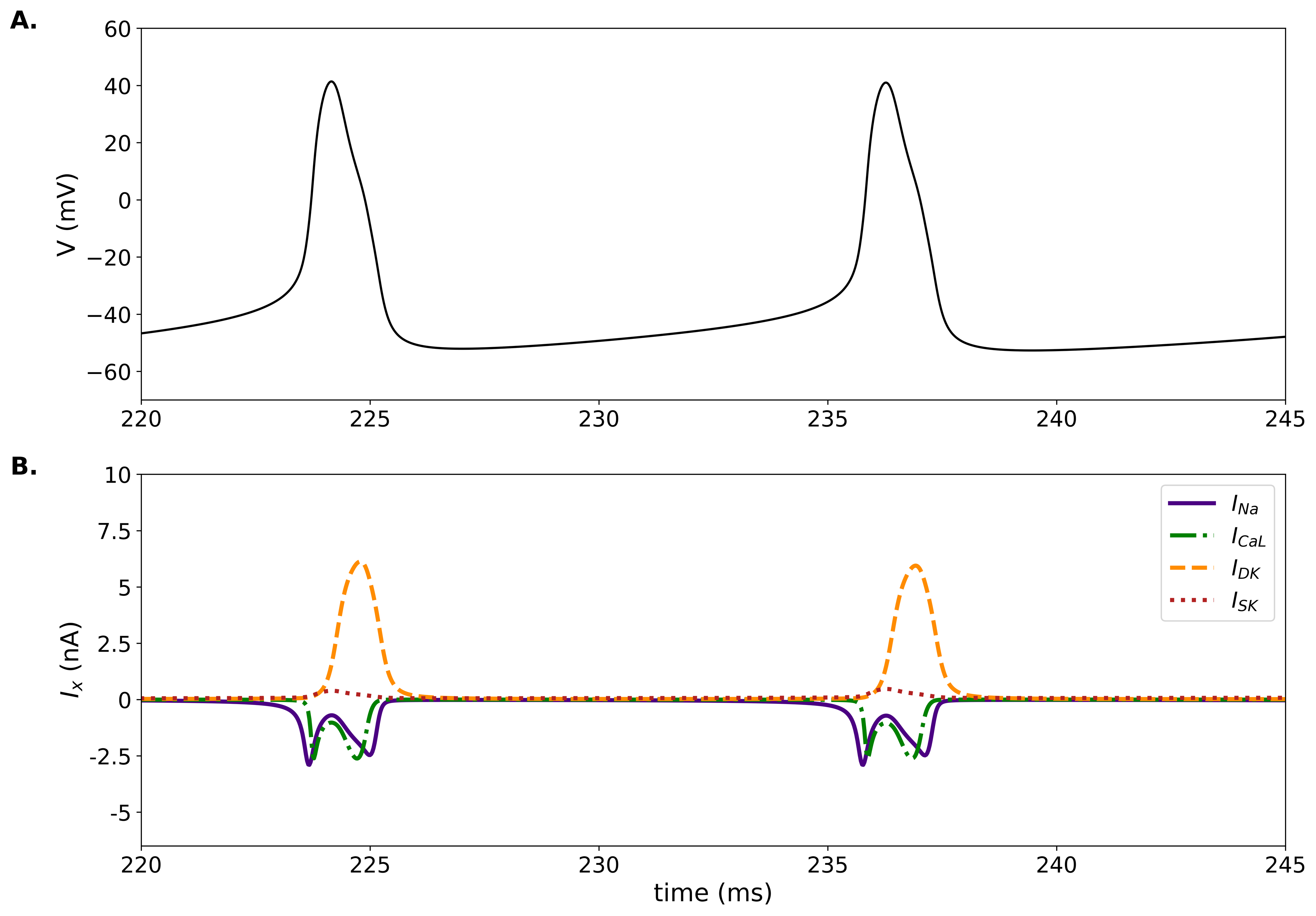

Supplement: S3 Fig — (A.) Two action potentials (APs) from the response seen in S2 Fig. (B.) Voltage- and Ca2+-gated currents in the models as indicated in the legend, and their amplitudes and dynamics during the APs. Note that the Na+-K+pump current is not plotted due to its small amplitude. (TIF) [file pone.0308809.s003.tif]

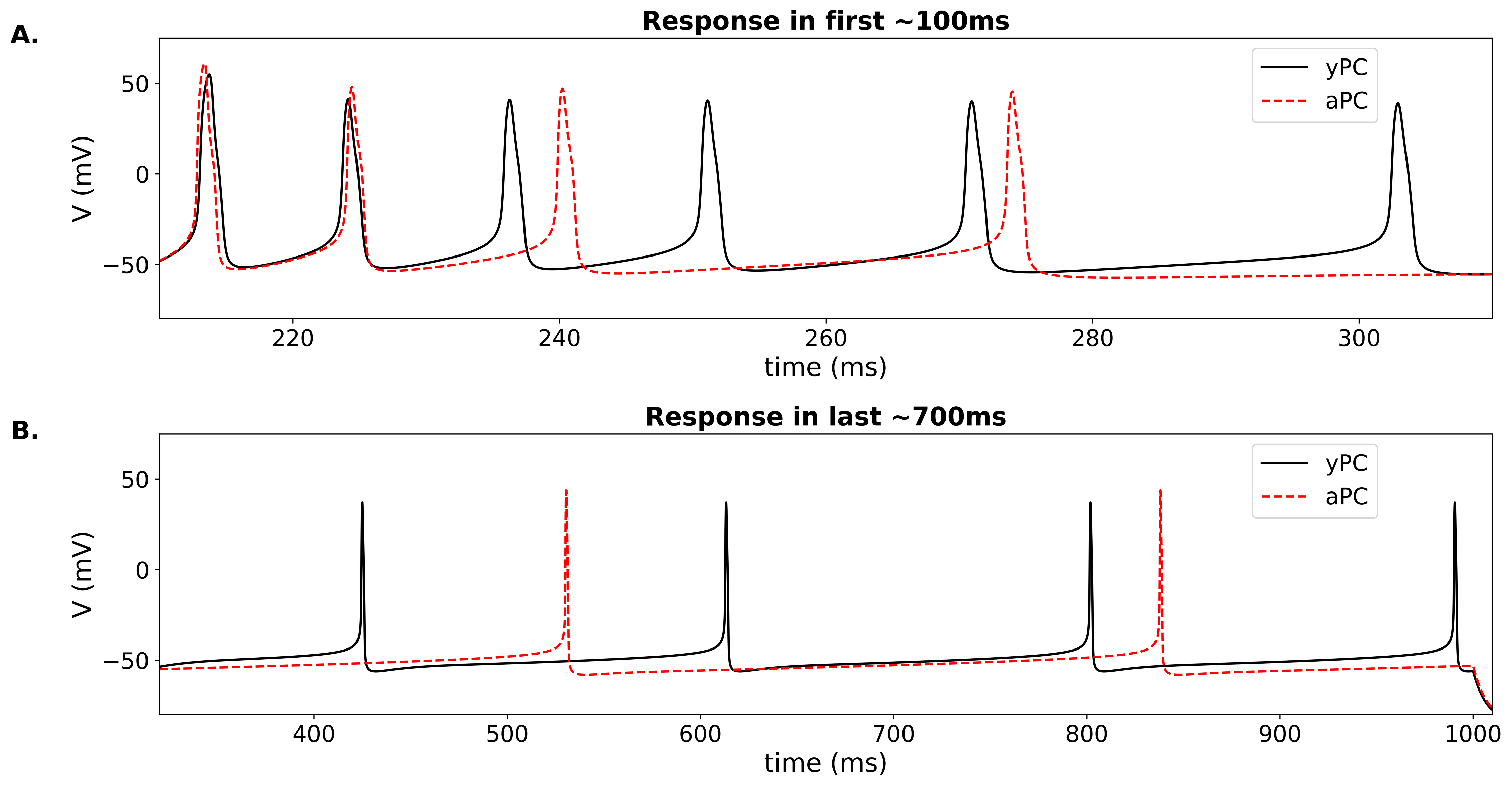

Supplement: S4 Fig — Further examination of the responses seen in Fig 1. (A.) Voltage responses of the yPC and aPC in the first ∼100 ms of the square-pulse stimulation (6 vs. 4 spikes). (B.) Voltage responses of the yPC and aPC in the last ∼700 ms of the square-pulse stimulation (4 vs. 2 spikes). (TIF) [file pone.0308809.s004.tif]

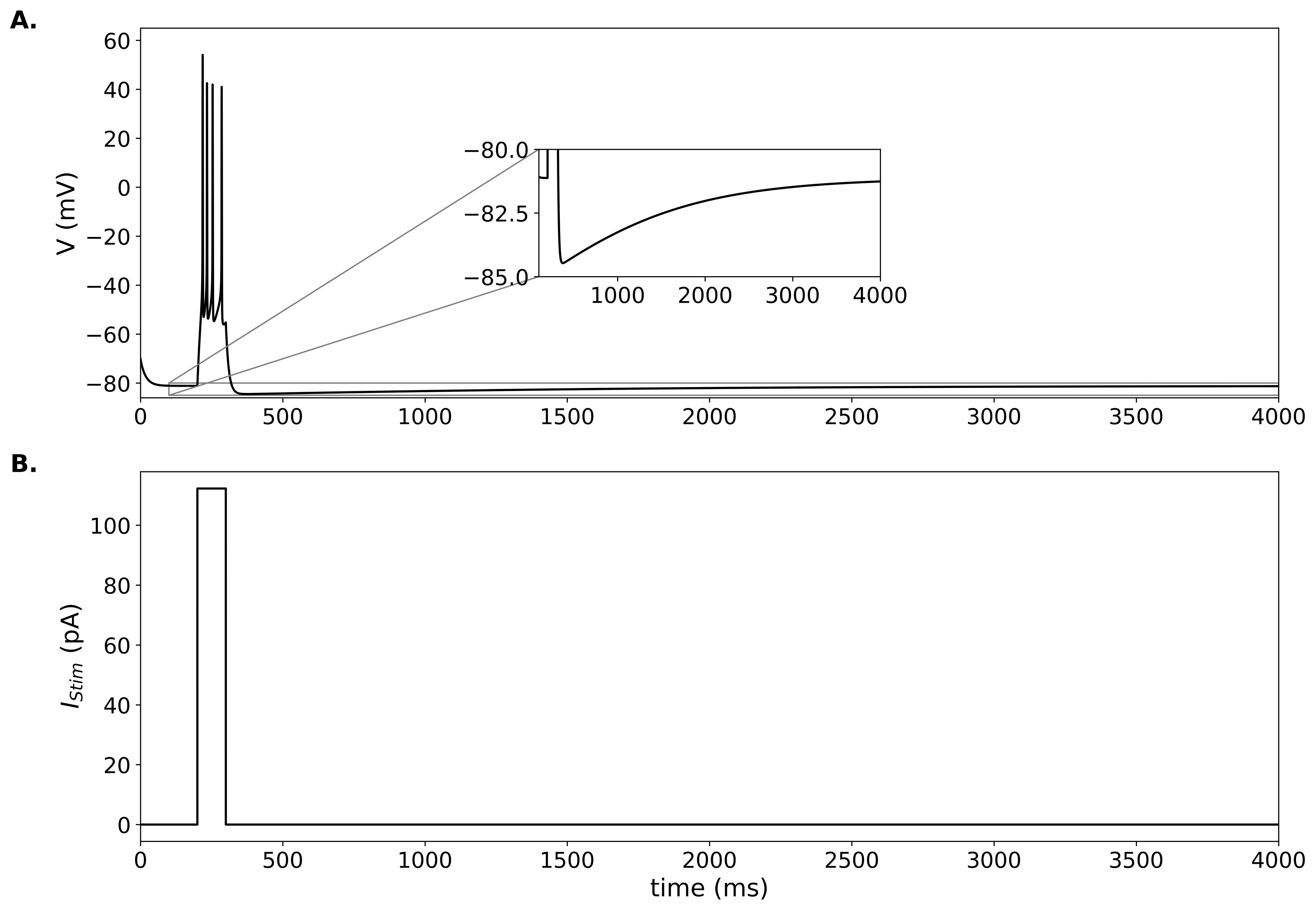

Supplement: S5 Fig — (A.) Voltage response to a 100 ms square-pulse current injection of sufficient amplitude to elicit 4 APs in the yPC. Inset shows the amplitude and duration of the resulting afterhyperpolarization (AHP). (B.) Current pulse. (TIF) [file pone.0308809.s005.tif]

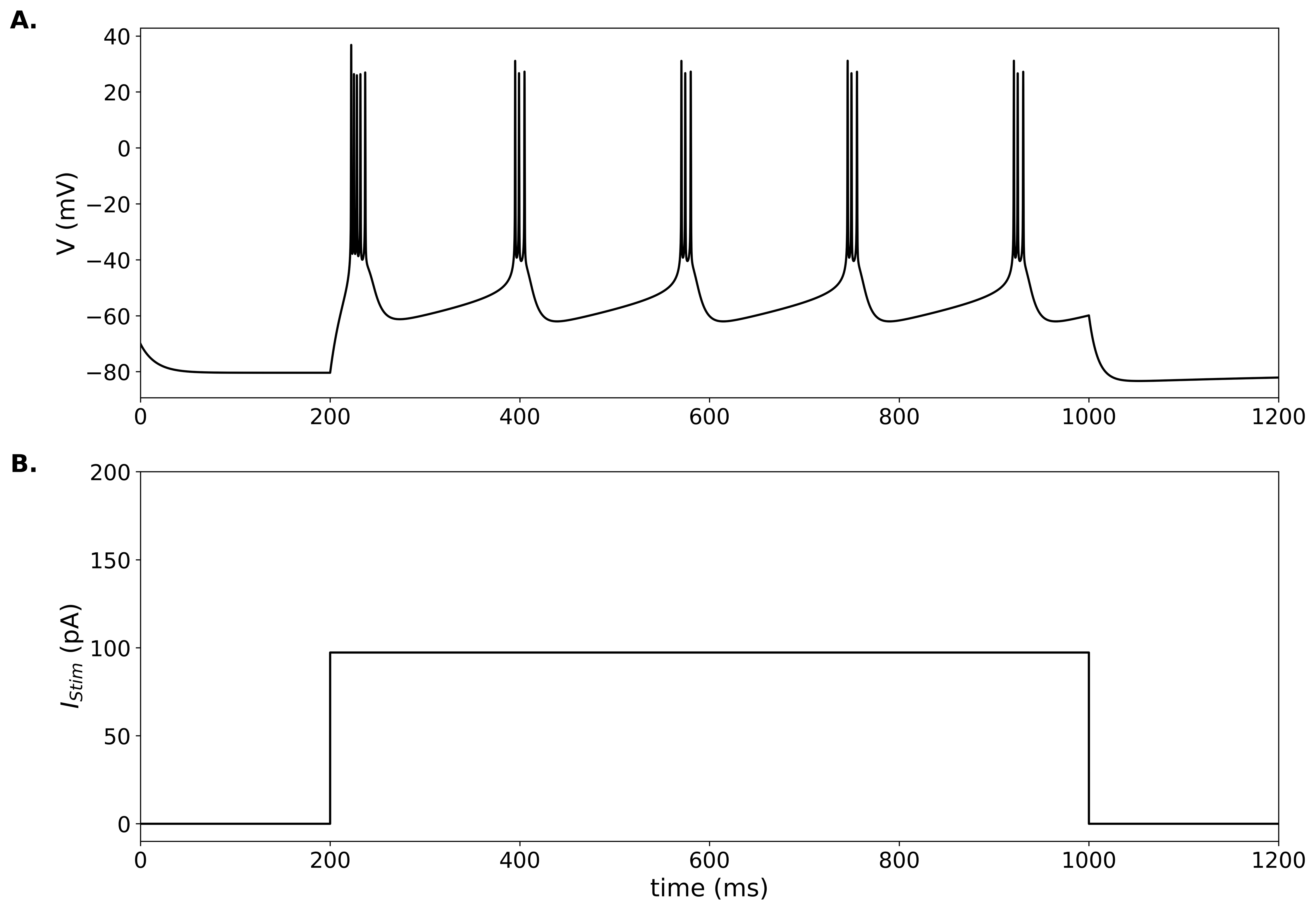

Supplement: S6 Fig — (A.) Bursting in the yPC in response to a 800 ms 100 pA square-pulse current injection seen in (B.). (TIF) [file pone.0308809.s006.tif]

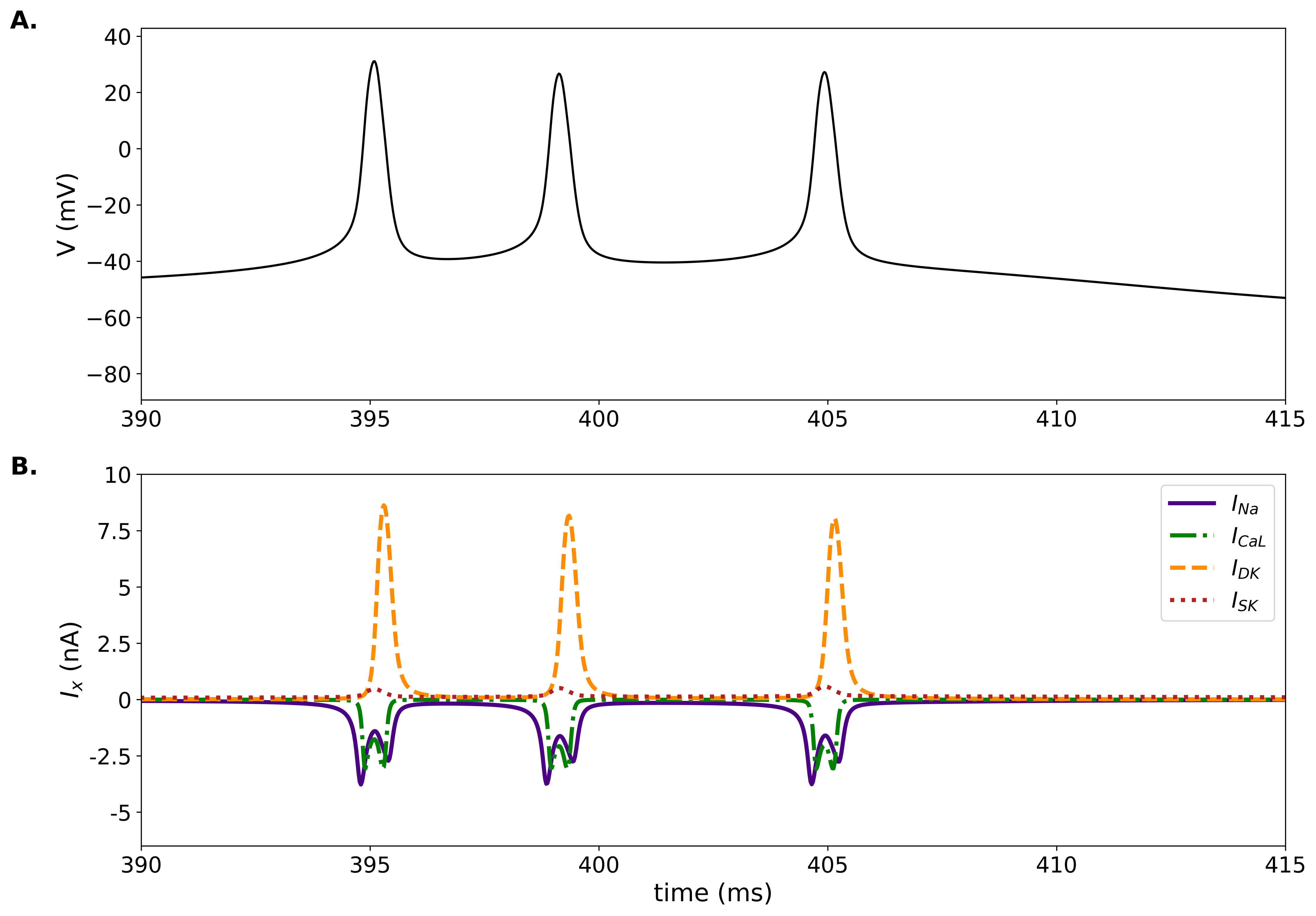

Supplement: S7 Fig — (A.) Three APs from the response seen in S5 Fig. (B.) Voltage- and Ca2+-gated currents in the models as indicated in the legend, and their amplitudes and dynamics during the APs. Note that the Na+-K+pump current is not plotted due to its small amplitude. (TIF) [file pone.0308809.s007.tif]

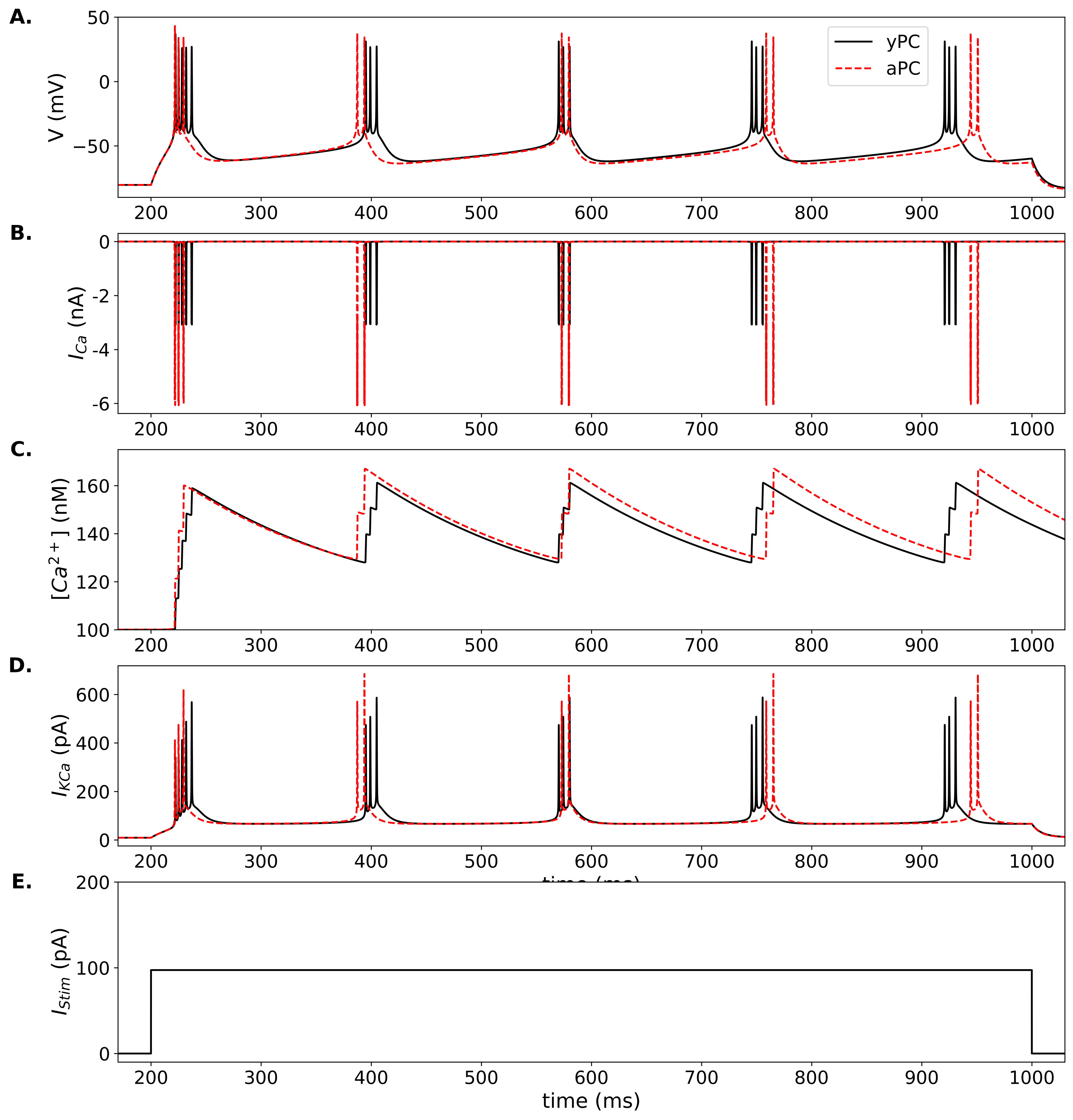

Supplement: S8 Fig — A. Bursting in the yPC (solid black traces) versus aPC (dashed red traces) in response to a 800 ms 100 pA square-pulse stimulation seen in (E.). Corresponding Ca2+ currents, intracellular Ca2+ concentration, and SK currents are shown in (B.), (C.), and (D.), respectively. (TIF) [file pone.0308809.s008.tif]

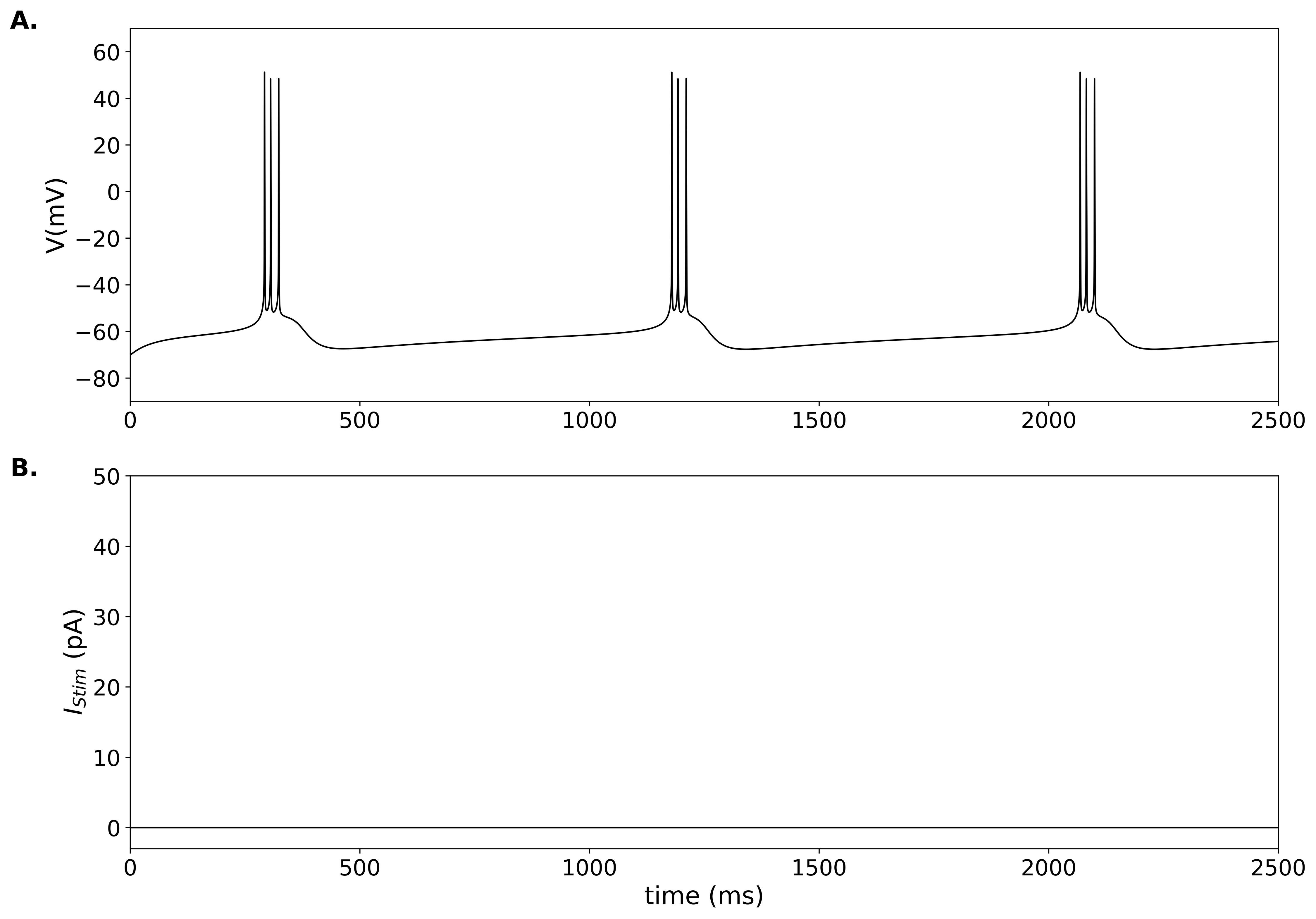

Supplement: S9 Fig — (A.) Changes in membrane potential (i.e. spontaneous bursting) in the absence of current stimulation (B.). (TIF) [file pone.0308809.s009.tif]

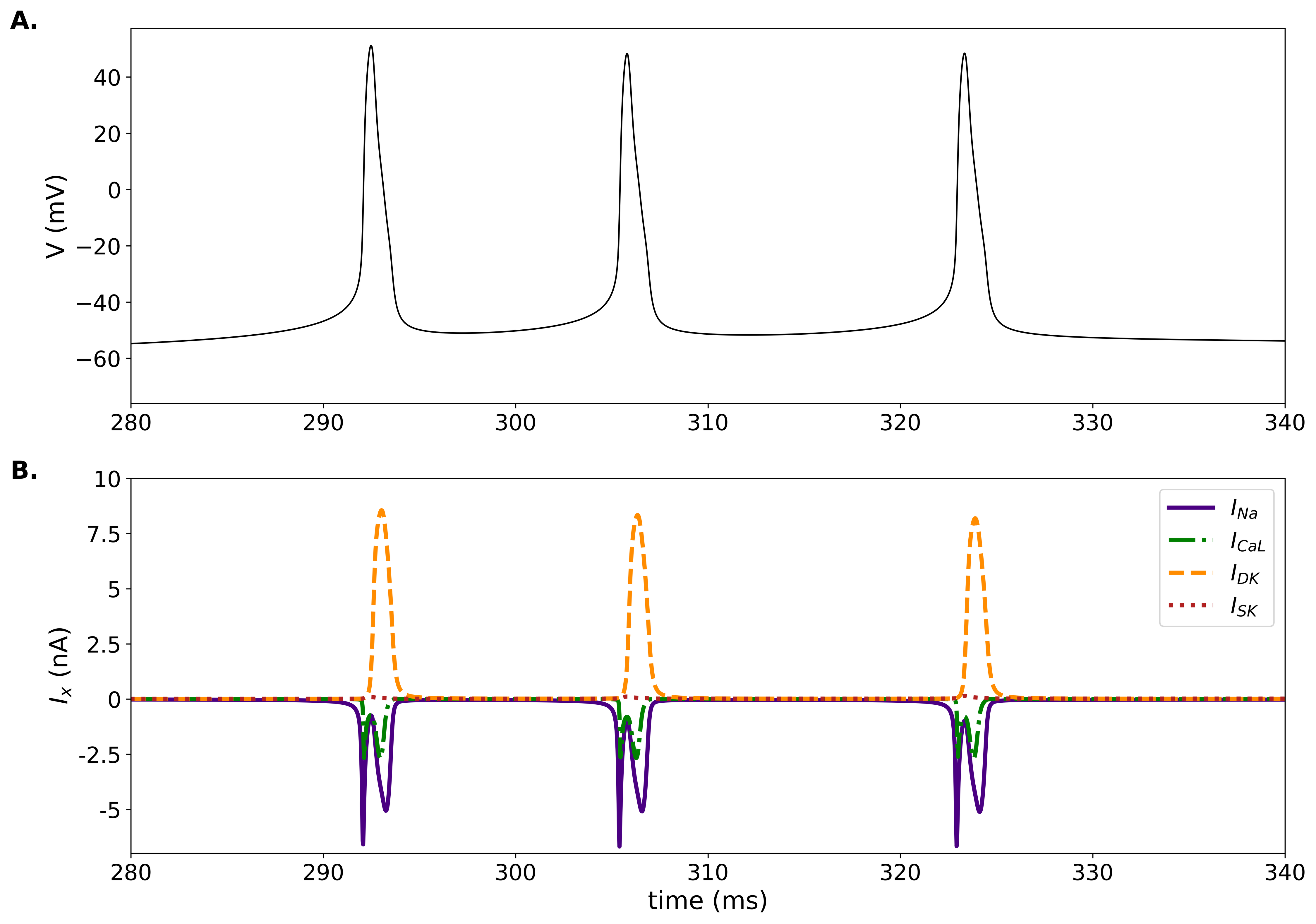

Supplement: S10 Fig — (A.) Three APs from the response seen in S8 Fig. (B.) Voltage- and Ca2+-gated currents in the models as indicated in the legend, and their amplitudes and dynamics during the APs. Note that the Na+-K+pump current is not plotted due to its small amplitude. (TIF) [file pone.0308809.s010.tif]

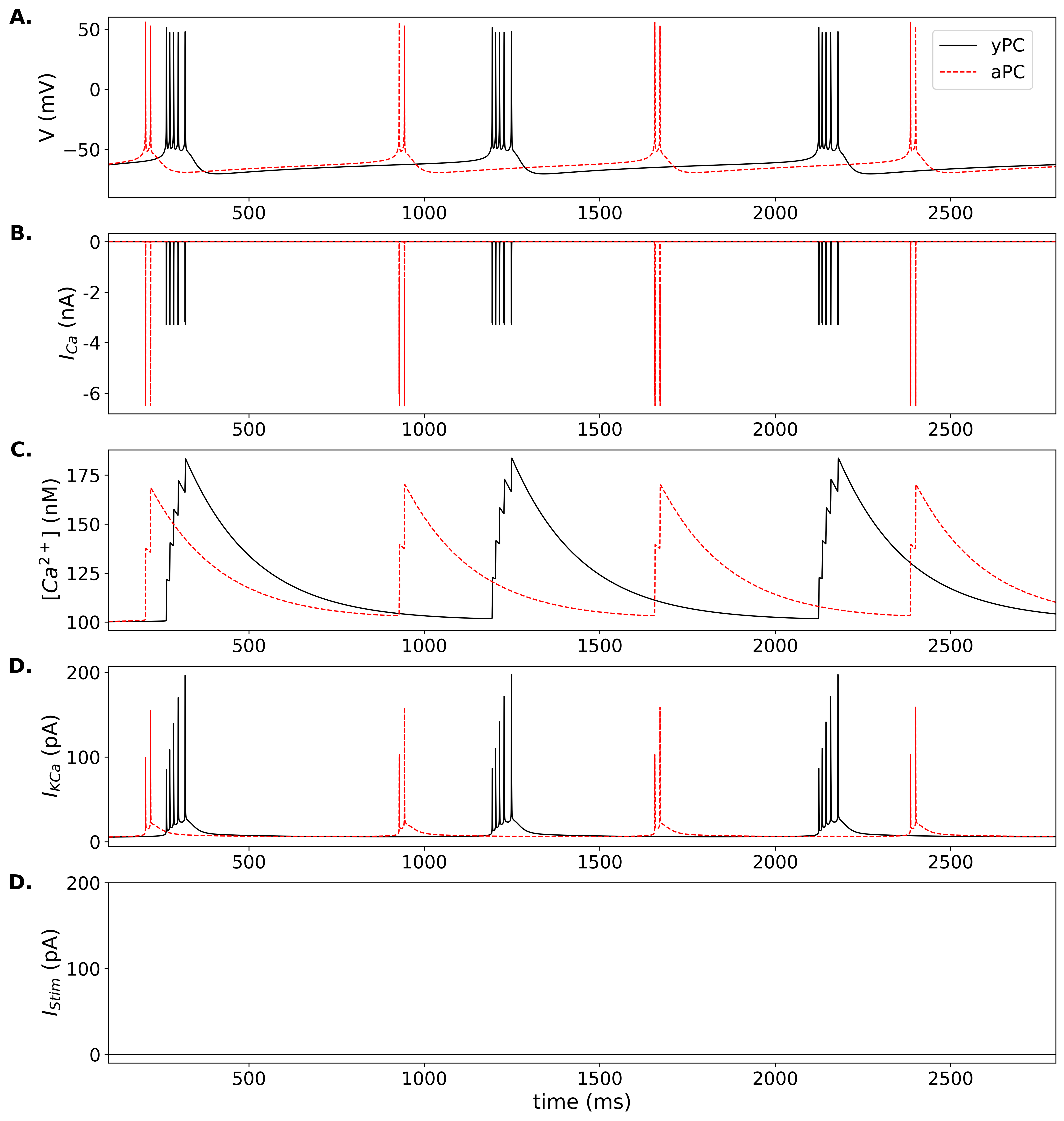

Supplement: S11 Fig — A. Bursting in the yPC (solid black traces) versus aPC (dashed red traces) in the absence of stimulation (E.). Corresponding Ca2+ currents, intracellular Ca2+ concentration, and SK currents are shown in (B.), (C.), and (D.), respectively. (TIF) [file pone.0308809.s011.tif]
