# Supplementary material for: A biophysical minimal model to investigate age-related changes in CA1 pyramidal cell electrical activity
Source: PLoS One. 2024 Sep 4;19(9):e0308809. doi: 10.1371/journal.pone.0308809 (PMC11373847; doi:10.1371/journal.pone.0308809)
Supplement: Attachment — Submitted filename: McKiernan_responseReviewers.pdf [file pone.0308809.s012.pdf]

23 July 2024

Manuscript ID: PONE-D-24-14388

Reviewers' Comments to the Author

Reviewer #1: The model presented in this study is impressive, particularly given its simplicity: with just three variables (though many constants), with only one parameter to reproduce the age-related differences. It effectively reproduces the differences between young and old CA1 pyramidal cells in the hippocampus, such as adaptive firing, stimulus-induced bursting, and spontaneous bursting. I thoroughly enjoyed reading it and congratulate the authors on their impressive results, along with the well-done Jupyter notebook that complements the paper excellently. I encourage the authors to continue their work by exploring bifurcation analysis and conducting a more robust exploration of the model's parameter limitations compared to experimental values.

I hope my comments can help to improve this paper.

We thank the reviewer for all their positive feedback, and suggestions for improving this paper. We also thank the reviewer for their encouragement regarding future work.

Major comments:

1) Abstract: The problem is well-introduced, and the rationale for the study is clear. However, please add some statements at the end about the conclusions of your research and its relevance to the field (what's the novelty?).

We added two closing sentences to the abstract, which talk about our novel results and summarize our conclusions regarding the potential power of this model in complementing experimental studies of aging (marked in blue; no line numbers).

2) In the introduction you pose some questions [3-8] that are not fully answered by the paper.
2.1) I'd consider revisiting this questions in the discussion together with a mention on how this work could help in the field of AD or Parkinson's, or other age-related pathologies.

We've added text to the Discussion section on 'Cellular heterogeneity', which we think better links the proposed experiments we had there previously to some of the questions we pose in the Introduction regarding normal neurophysiological aging and its stages (lines 362-367 and lines 370-373). We also added some text therein and an additional reference regarding how this model might be used to help understand aspects of Alzheimer's disease (lines 366-367, ref. 93).

2.2) Can this be used to model the impairment of plastic mechanisms?

The model in its current formulation cannot be used to study plasticity, since it does not include equations to model synaptic input or neurotransmitter release. However, the model could be extended to do this, which we would like to explore in future work.

3) Methods [46-56]: Clarify better what distinguishes this model from the one previously developed.

We added language to the Methods section to clarify that this present formulation is different from our previous formulations in that it includes calcium dynamics (i.e. is three-dimensional

rather than two-dimensional), and is also specially tuned with parameter values taken from experimental data from hippocampal PCs (lines 55-59).

4) Methods Section 2.1: Please provide a brief explanation of what I_{F} and I_{CaL} are when explaining Equation 1.

We've added these brief explanations (lines 72-75).

5) Clarification on s_x and N_x: The values of s_x are unclear for non-voltage-gated channels (NaT adn DK?), together with the values of N_x. Can you consider adding this information to the Table 1? Is there supporting literature from the values you choose?

Regarding the values of s_x, we write, "s_x is ~1 pA for most voltage-gated channels [38], and is ~5-10 pA for SK channels" (lines 90-91), and we cite two references from the literature that support these values (refs. 38, 39). The number of channels in the membrane, N_x, for each of these channels is not well known in CA1 PCs, to the best of our knowledge. We do not enter values for s_x or N_x directly as parameters in the model, which is why they are not included in the parameter table (Table 2). If these are well known in other cells, they could be entered, but given that we do not have good numbers for the second, what we instead enter into the model as a parameter is the amplitude, a_x, which is the product of s_x and N_x. These amplitudes are chosen to produce currents of the same amplitude as seen in experimental recordings, and the references from the literature supporting those values are included in Table 2.

6) Consider adding the figures of the Jupyter notebook in an Appendix/Supporting Material, so that it is easier to reference. They will help understanding the model functions and variables, contributions of I_x to V...

We've added all the additional figures generated in the Jupyter notebook to the manuscript as Supporting Information (Figs S1-S11).

7) Recent paper of potential interest to add to the paper (bursting patterns aged vs. young, line 168-169): https://www.ncbi.nlm.nih.gov/pmc/articles/PMC10926450/ also consider mentioning it in the intro/discussion.

We had missed this paper, thank you for flagging. We've made a small change to the Study Design section (what was lines 168-169 and is now lines 211-213), adding the word 'their' to specify that here we mean we are not aware of any studies that look at the bursting patterns of CA1 PCs in young and aged animals. From reading the above paper, we see that they did look at bursting in VTA cells but not in hippocampal cells, where they were more focused on synaptic plasticity. However, the comparison of young and aged VTA cells is interesting and relevant, and so we added a brief description of this study's results to the Discussion section (lines 446-452).

8) General comments to the figures:

8.1 There are no captions (?);

Our apologies for this omission. We corrected this and there are now captions for all figures.

8.2 Please add the legend (yPC, aPC) + see next comment

We've added legends to all figures.

9) Figure 1 (and methods 3.1).

9.1 (top panel) Not clear how do you compute the frequency (e.g., 60Hz vs. 40Hz). I think I'd be helpful to have in the figure a visual separation between the two segments you are referring to

(first 100ms + the rest), plus a bar plot (for example) that shows quantitatively the differences between young and aged PCs.

The frequency is calculated manually by simply counting the number of spikes in the given time window, e.g in the initial 100 ms we see 6 vs. 4 spikes, which comes out to 60 Hz and 40 Hz, respectively. To aid in comparing the two segments (first 100 ms vs. last 700 ms) we added a supplemental figure in the Jupyter notebook and in Supporting Information (S4 Fig), and added reference to this figure in this section of the main text (lines 242-244).

We think a bar plot might be misleading, since it could imply we have more than one data point (more than one 'subject') for the young and aged PCs. Since this is a deterministic model, running these simulations with the same parameters always produces the same result.

9.2 Also, If you don't want to include the figures in the notebook, at least add in the plot when the square pulse finishes and ends and with which amplitude.

We've added all the additional figures generated in the Jupyter notebook, including those that show the stimulus traces, to the Supporting Information. We've also added the stimulus traces to the main figures within the manuscript, with the exception of two figures where the stimulus amplitude varies (Fig. 3) or there is no stimulus (Fig. 4).

9.3 Again, I'd add the rest of the figures (which are relevant in my opinion) in the appendix (at least).

We've added all the additional figures in the Jupyter notebook to the Supporting Information. We've also added references to these supplemental figures in the text, where relevant.

9.4 Rephrase the text 197-201 so that it is easier to understand which panel you are referring to.

We appreciate the reviewer pointing this out. We realized this was a bit confusing for all figures (not just this section). So, we've redone all figures with A, B, C, etc. labels for each panel and now refer to the panels this way throughout the text (each instance marked in blue).

9.5 All above (9.1-9.3) also applies to the other figures (when applicable)!!

We've applied these recommendations to all figures, as applicable.

10) Parameter tuning: In the methods (and/or results), explain how parameters are tuned to find each firing pattern (I understand it is not only based on literature). Clarify which parameters are changed for each plot.

We added language to the Methods section to explain how the parameters were tuned (lines 177-188). Regarding clarifying which parameters are changed, we thank the reviewer for this prompt. Changes to the parameters are now indicated in all the figure captions.

11) Figure 2, Bottom Panel: Indicate whether the graph shows saturation or continuous increase. If not changing the plot, mention in the caption that this is not a saturation effect.

This is not a saturation effect or a continuous increase, but rather shows the AHP response after a short stimulus, and then that response gradually running down over time after the stimulus ends. We think this is clearer now that we have added the stimulus pulse to the plot, and we thank the reviewer for that suggestion for all figures.

12) Figures 5 and 6: Consider merging these figures as they contain overlapping information. Clearly indicate which parameters are changed between plots and the reasons for those changes.

We think both of these figures are important, since they show results for different firing regimes and reproduce different electrical phenomena seen in PC recordings. So, we have kept these figures. The parameters used for each plot are indicated by referring to parameters in Figs. 1 and 5. The only LFP parameter changed between the two plots is indicated in the Fig. 6 caption.

Minor comments:

31 means → a means

Corrected. In the current template, this is now on line 35.

46 CA1 in blue?

Corrected.

There is a problem with the references to the figures, and It was very hard to guess which figure you might be referring to.

Our apologies, we had LaTeX compilation errors that meant the figure numbers were not displayed. We have fixed these and all figure references should now be correct.

Remove paragraph space between 290-291.

This is a new paragraph (now on line 357).
